# Longitudinal profiling of oligomeric Aβ in human nasal discharge reflecting cognitive decline in probable Alzheimer’s disease

**DOI:** 10.1038/s41598-020-68148-2

**Published:** 2020-07-08

**Authors:** Seung-Jun Yoo, Gowoon Son, Jisub Bae, So Yeun Kim, Yong Kyoung Yoo, Dongsung Park, Seung Yeop Baek, Keun-A Chang, Yoo-Hun Suh, Yeong-Bae Lee, Kyo Seon Hwang, YoungSoo Kim, Cheil Moon

**Affiliations:** 1 Department of Brain and Cognitive Sciences, Graduate School, Daegu Gyeungbuk Institute of Science and Technology, Daegu, Republic of Korea; 2 Convergence Research Advanced Centre for Olfaction, Daegu Gyeungbuk Institute of Science and Technology, Daegu, Republic of Korea; 3 Department of Clinical Pharmacology and Therapeutics, College of Medicine, Kyung Hee University, Seoul, Republic of Korea; 4 Integrated Science and Engineering Division, Department of Pharmacy, Yonsei Institute of Pharmaceutical Sciences, Yonsei University, Incheon, Republic of Korea; 5 Department of Pharmacology, School of Medicine, Gachon Medical School, Incheon, Republic of Korea; 6 Department of Neurology, Gil Medical Center, Gachon University, Incheon, Republic of Korea

**Keywords:** Diseases of the nervous system, Olfactory system

## Abstract

Despite clinical evidence indicating a close relationship between olfactory dysfunction and Alzheimer’s disease (AD), further investigations are warranted to determine the diagnostic potential of nasal surrogate biomarkers for AD. In this study, we first identified soluble amyloid-β (Aβ), the key biomarker of AD, in patient nasal discharge using proteomic analysis. Then, we profiled the significant differences in Aβ oligomers level between patient groups with mild or moderate cognitive decline (n = 39) and an age-matched normal control group (n = 21) by immunoblot analysis and comparing the levels of Aβ by a self-standard method with interdigitated microelectrode sensor systems. All subjects received the Mini-Mental State Examination (MMSE), Clinical Dementia Rating (CDR), and the Global Deterioration Scale (GDS) for grouping. We observed higher levels of Aβ oligomers in probable AD subjects with lower MMSE, higher CDR, and higher GDS compared to the normal control group. Moreover, mild and moderate subject groups could be distinguished based on the increased composition of two oligomers, 12-mer Aβ*56 and 15-mer AβO, respectively. The longitudinal cohort study confirmed that the cognitive decline of mild AD patients with high nasal discharge Aβ*56 levels advanced to the moderate stage within three years. Our clinical evidence strongly supports the view that the presence of oligomeric Aβ proteins in nasal discharge is a potential surrogate biomarker of AD and an indicator of cognitive decline progression.

## Introduction

Alzheimer’s disease (AD) is the most common type of dementia characterized by progressive cognitive decline and the accumulation of the both amyloid-β (Aβ) plaques and tau neurofibrillary tangles in the brain. The diagnosis of AD requires pathological observations in the central nervous systems^1–7^. However, current diagnostic methods are not for routine measurements to consider the reliability of diagnosis. In addition, accessing brain tissues and cerebrospinal fluid is invasive and positron emission tomography exposes subjects to radioactive tracers. Although blood is considered one of the most promising fluid biomarker candidates for AD, this approach still has to overcome critical issues, such as low concentrations (e.g. p-tau: ~ pg/ml)^8^, plasma stability, blood–brain barrier penetration of key protein biomarkers to be used for primary diagnosis and prognosis.

Fluid biomarkers within the peripheral nervous systems could be an excellent solution to avoid limitations utilizing cerebrospinal fluid and blood. The olfactory system, in particular the olfactory epithelium, has unique amyloid precursor protein (APP)—processing mechanisms. The olfactory epithelium (OE) has unusual secretases expression compare to CNS, and distinct increased expression of presenilin 1 and 2 (γ-secretase) under pathological conditions^9^. Olfactory dysfunction is often observed concurrently with or prior to cognitive impairment in AD and other dementia based on epidemiological evidence (~ 90%)^10–12^. In particular, the high prevalence of olfactory dysfunction among AD patients indicates a possible correlation between olfactory deficits and AD pathogenesis (i.e., the expression of Aβ in the olfactory tissue)^13–16^.

Based on this clinical and experimental evidence, we hypothesized that nasal discharge could be a candidate to monitor pathophysiological changes in the olfactory system during neurodegeneration that may result in AD. Our previous animal study supports the hypothesis by reporting that specific oligomeric Aβ (Aβ*56 and AβO) was existed in the olfactory epithelium at different progression stages of AD with evident cognitive impairment in AD transgenic mice; Tg2576, a Swedish mutant form of human amyloid precursor protein (APP) (KM670/671NL) (promoter: hamster prion protein (PrP))^16^. In addition, there are previous studies that prove that there is a direct linkage pathway between CSF and olfactory systems that directly reflect alterations occurring in the CNS, and there are also previous studies in which biomarkers of CSF are found in olfactory mucosa. Taking all possibilities together, nasal discharge may contains a wide assortment of proteins released from damaged olfactory sensory neurons or outflow of CSF through the cribriform plate under pathological condition^17–19^.

In this study, we obtained nasal discharge samples from both the probable AD group and the age-matched normal control group and examined the presence of Aβ in the nasal discharge and determined the type of Aβ oligomers specific to the patient group. Then, we demonstrated that Aβ was expressed in the nasal discharge from AD patients by liquid chromatography-mass spectrometry (LC–MS) and that changes in the oligomeric Aβ composition in the nasal discharge were correlated with cognitive decline among the patient groups by immunoblot analysis. We also identified the expression patterns of oligomeric Aβ species in patients with different stages of cognitive dysfunction by immunoassays and comparing the levels of Aβ by a self-standard (CLASS) method to measure the self-standard ratio defined the value of the impedance change in the monomerized sample divided by intact sample with interdigitated microelectrode (IME) sensor systems^20^. Lastly, we assessed alterations in two specific types of oligomeric Aβ levels in nasal discharges in three-year longitudinal cohorts.

## Results

### Cohorts

We recruited participants and grouped them into clinically confirmed cohorts. Subjects were categorized according to the National Institute of Neurological and Communicative Disorders and Stroke and the Alzheimer’s Disease and Related Disorders Association (NINCDS/ADRDA) of the American Psychiatric Association criteria for the diagnosis of probable AD^1,21–23^. A total of 60 participants were enrolled, 21 in the normal control group and 39 in the probable AD group (Table 1)^23^. Statistically significant differences between the groups were detected in the Mini-Mental State Examination (MMSE) (dementia: 20.47 ± 3.73; normal: 27.61 ± 0.85, *P* < 0.001) and the Clinical Dementia Rating (CDR) scores (dementia: 0.65 ± 0.23; normal: 0.47 ± 0.12, *P* < 0.001) (Table 1). Moreover, the Global Deterioration Scale (GDS) scores of the dementia group was significantly higher than the normal group (dementia: 3.29 ± 0.37; normal: 1.94 ± 0.24, *P* < 0.001) (Table 1).

**Table 1:** Summary of cognitive assessments and age by disease status and gender.

Conditions	Normal		Dementia (probable AD)	
Gender	Male	Female	Male	Female
Number of subjects	21		39	
	10	11	24	15
Age (years; mean ± SE)	71.92 ± 5.27		76.30 ± 6.18 (ns)	
	73.72 ± 6.92	70.29 ± 3.77	76.92 ± 6.84	75.32 ± 5.12
MMSE (mean ± SE)	27.61 ± 0.85		20.47 ± 3.73 (***)	
	27.55 ± 0.82	27.71 ± 0.95	21.62 ± 3.43	19.88 ± 0.63
CDR (mean ± SE)	0.47 ± 0.12		0.65 ± 0.23 (***)	
	0.45 ± 0.15	0.50 ± 0.00	0.61 ± 0.22	0.66 ± 0.24
GDS (mean ± SE)	1.94 ± 0.24		3.29 ± 0.37 (***)	
	2.00 ± 0.00	1.85 ± 0.38	3.31 ± 0.63	3.36 ± 0.57

The data are presented as means ± SEs. For the statistical analysis, one-way ANOVA was performed, followed by Dunnett’s post-hoc test. Statistical significance is denoted as ns: *P* > 0.05, ***P < 0.001. MMSE, CDR, and GDS refer to the Mini-Mental State Examination, the Clinical Dementia Rate, and the Global Deterioration Scale, respectively.

### Identification of oligomeric Aβ in nasal discharge

**Figure 1:**
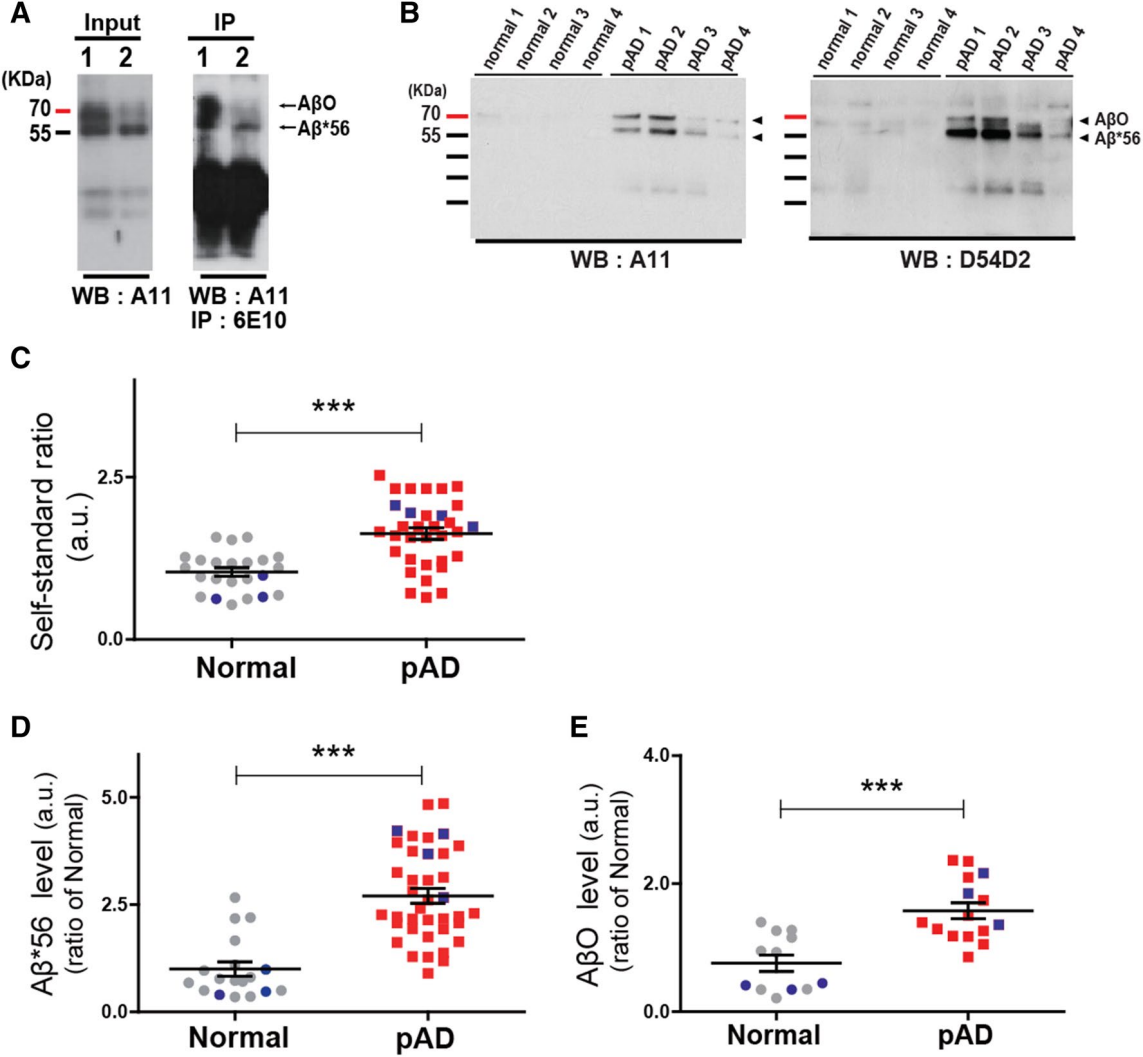
Soluble Aβ oligomers are detected in the nasal discharges from probable AD group (pAD). Immunoblotting verification; soluble Aβ oligomers were detected in the nasal discharges of pAD group. (**A**) Identification of Aβ oligomer, assessed by western blot (WB; A11) with or without immunoprecipitation (IP: 6E10) using samples (1; pAD1 and 2; pAD2) from pAD group. (**B**) Representative data for A11-immunoreaactive (left) and D54D2-immunoreaactive (right) soluble Aβ oligomers are detected in nasal discharges of pAD group (4; pAD1, pAD2, pAD3 and pAD4) and normal group (4; normal 1, normal 2, normal 3 and normal 4). (**C**) The total levels of soluble Aβ species in nasal discharges were measured between the normal and pAD groups using CLASS method (self-standard ratio (a.u.)). (**D**) Quantification of soluble Aβ*56 protein. Expression levels of proteins were quantified using stereological analysis (ImageJ program). (**E**) Quantification of soluble AβO protein. Expression levels of proteins were quantified using stereological analysis (ImageJ program). Data are represented as means ± SEMs from three independent experiments. Value of samples were identified as outliers through Grubbs’ test, also called the ESD method. For statistical analysis, paired t-test was performed. Statistical significances are denoted (***P < 0.001).

We first examined the presence of Aβ monomers and oligomers in the nasal discharge samples of two probable AD subjects. We performed LC–MS/MS analysis using samples immunoprecipitated with an antibody against an Aβ peptide (6E10, Covance, Princeton, NJ, USA). A 12-residue internal tryptic peptide sequence of LVFFAEDVGSNK, identical to human Aβ (17–28), was detected. Because various forms of distinct Aβ are deposited in spatially and temporally distinct manners during AD progression, we examined the specific soluble oligomerized Aβ species in nasal discharges using immunoblot analysis. We performed an immunoblot assay of nasal discharges from the possible AD group using an antibody against various Aβ oligomers (A11, Invitrogen, Carlsbad, CA, USA) after immunoprecipitation with 6E10. We identified assemblies of Aβs, Aβ*56, 12-mer peptide (~ 56 kDa), and AβO, 15-mer peptide (~ 80 kDa), in the nasal discharge from the probable AD group (Fig. 1A). Next, we measured levels of Aβ*56 and AβO in the nasal discharge samples of normal controls and probable AD subjects by an immunoblot assay with an anti-oligomeric A11 antibody and an anti-Aβ D54D2. Compared to the normal control group, the probable AD group exhibited the increased expression of both sizes of oligomeric Aβ in their nasal discharges (Fig. 1B).

### Increased oligomeric Aβ in the nasal discharge of the probable AD group

To verify the presence of upregulated soluble Aβ oligomers in the probable AD group, we used our previously verified CLASS method with IME sensor systems^20^. In our previous research, the CLASS method demonstrated high accuracy in discerning the normal group from the AD group in human plasma. The CLASS method dissociates aggregated Aβ into monomers by a chemical, EPPS ([4-(2-hydroxyelthyl)-1-piperazinepropanesulfonic acid]), and allows quantitative measurements of oligomeric Aβ in proportion to the total Aβ pool in nasal discharge samples. The IME sensor system is a highly sensitive electrical detection tool to identify the presence of protein biomarkers at sub pg/mL scales. The integration of the CLASS method along with a highly sensitive IME sensor system enabled us to avoid the individual fluctuations that can occur from the conventional analysis of amyloid aggregates under heterogeneous conditions. Conventional methods measuring Aβ levels under the presence of various Aβ conformations were not successful, which led to the misinterpretation of Aβ as an unreliable biomarker^24–26^. The self-standard ratio obtained from both the normal control group and the probable AD group was used to assess the levels of oligomeric Aβ in the nasal discharge. The levels of oligomeric Aβ species in the nasal discharge of the probable AD group (n = 39) were significantly higher than those of the normal control group (n = 21) (Fig. 1C).

The levels of Aβ*56 and AβO in the nasal discharge samples were also measured by immunoblot analysis and we found higher expression of both oligomeric Aβs in the probable AD group compared to the normal control group (Fig. 1D, E). When we examined the correlations between the Aβ oligomer levels and other non-AD factors (e.g., age and sex), there was no correlation between Aβ oligomer levels and age and sex (Fig. S2). Taken together, our results suggest that the detection of soluble Aβ oligomers in the nasal discharge could be a specific feature of probable AD patients.

### Different profiles of Aβ*56 and AβO in AD stages

**Figure 2:**
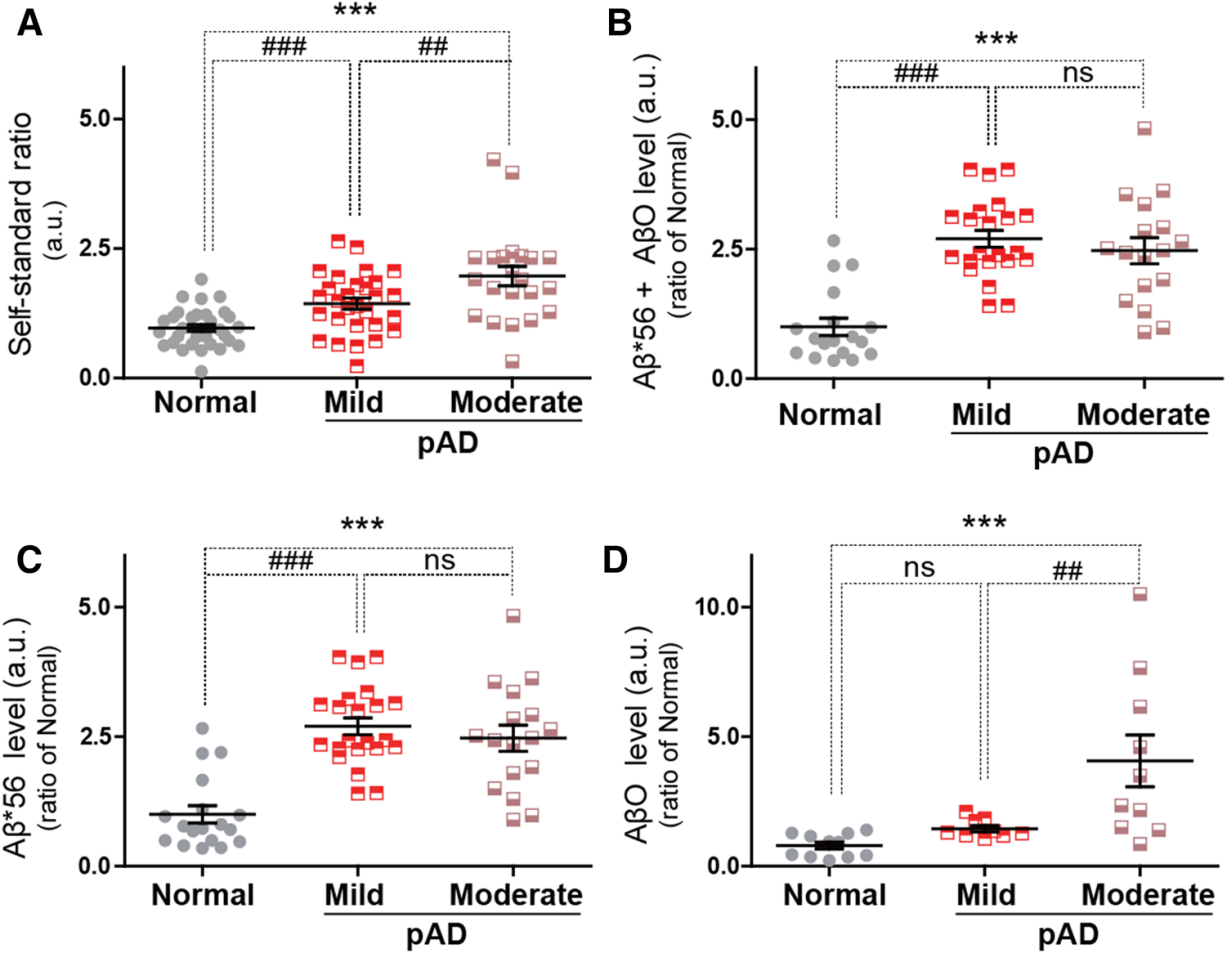
The specific composition of soluble Aβ in nasal discharges from different stages of probable AD group (pAD). (**A**) The total levels of soluble Aβ species using CLASS method (self-standard ratio (a.u.)) in nasal discharges were measured in (Normal (22), mild stage (25) and moderate stage (13) of pAD groups. (**B**) Quantification of soluble Aβ*56 + AβO protein levels. Expression levels of proteins were quantified using stereological analysis (ImageJ program) in Normal (18), mild stage (22) and moderate stage (17) of pAD groups. (**C**) Quantification of soluble Aβ*56 protein. Expression levels of proteins were quantified using stereological analysis (ImageJ program) in Normal (18), mild stage (22) and moderate stage (17) of pAD groups. (**D**) Quantification of soluble AβO protein. Expression levels of proteins were quantified using stereological analysis (ImageJ program) in Normal (12), mild stage (9) and moderate stage (10) of pAD groups. Data are represented as means ± SEMs from three independent experiments. Value of samples were identified as outliers through Grubbs’ test, also called the ESD method. For the statistical analysis, one-way ANOVA was performed, followed by Dunnett’s post hoc test. Statistical significance is denoted (ns > 0.05, ***P < 0.001). In addition, paired t-test was performed. Statistical significances are denoted (ns > 0.05, ^#^*P* < 0.05, ^##^*P* < 0.01, ^###^*P* < 0.001).

The probable AD group was further categorized based on their MMSE, CDR, and GDS scores into mild (n = 26) and moderate stages (n = 13) as summarized in Table 2^1,21^. Compared to the normal group, the sum of the Aβ*56 and AβO levels was significantly higher in both groups and different between the two stages (Fig. 2A, B). The mean Aβ*56 levels were significantly higher in both the mild and moderate probable AD groups than the normal group, whereas we detected no significant difference between the mild and moderate AD groups (Fig. 2C). In contrast, the mean AβO levels were significantly higher in the moderate stage probable AD group than in the other two groups, whereas no significant difference was found between the normal and mild AD groups (Fig. 2D). Taken together, the Aβ oligomerization profiles in nasal discharge may vary depending on the AD progression, shown by the higher expression of Aβ*56 in the mild stage and higher expressions of both Aβ*56 and AβO in the moderate stage compared to the normal group.

**Table 2:** Summary of cognitive assessments and age by disease progression status.

Conditions	Normal	Dementia (probable AD)	
		Mild stage	Moderate stage
Number of subjects	21	26	13
Age (mean ± SE) (years)	72.39 ± 6.01	75.17 ± 5.61 (ns)	76.93 ± 5.92 (ns)
MMSE (mean ± SE)	27.61 ± 0.85	23.17 ± 1.11 (***)	16.33 ± 2.12 (***)
CDR (mean ± SE)	0.47 ± 0.12	0.52 ± 0.10 (ns)	0.83 ± 0.24 (***)
GDS (mean ± SE)	1.94 ± 0.24	3.04 ± 0.21 (***)	3.80 ± 0.68 (***)

Disease progression status was divided into three groups: normal (MMSE > 25, CDR < 0.5, and GDS < 2), mild (20 < MMSE < 25, 0.5 < CDR < 1, and 2 < GDS < 3), and moderate (MMSE < 20, CDR > 1, and GDS > 3). The data are presented as means ± SEs. For the statistical analysis, one-way ANOVA was performed, followed by Dunnett’s post-hoc test. Statistical significance is denoted as ns: *P* > 0.05, ***P < 0.001). MMSE, CDR, and GDS refer to the Mini-Mental State Examination, the Clinical Dementia Rate, and the Global Deterioration Scale, respectively.

These results suggest that profiling alterations of Aβ*56 and AβO levels in nasal discharge may distinguish the stages of AD-associated cognitive decline. We compared the oligomer proportion of total Aβ (Fig. S3A), the levels of Aβ*56 (Fig. S3B), and the levels of AβO (Fig. S3C) with MMSE scores (Table 3). Our results revealed that the self-standard ratio (a.u.) for the levels of oligomeric Aβ species moderately (0.6 > R > 0.4) correlated across the full range of MMSE scores (R = 0.5293; black line; Fig. S3A and Table 3). However, when stratified by MMSE scores, the levels of Aβ*56 better (R > 0.6) correlated (R = 0.6069; red line) with the mild AD and normal groups compared to the moderate AD group (R = 0.6815; pink line; Fig. S3B and Table 3). According to our results, the expression level of Aβ*56 in the nasal discharge was most correlated with cognitive function in the mild AD stage. In contrast, AβO levels strongly correlated (R = 0.6061; pink line) with moderate AD stage, and correlated (R = 0.4010; red line) slightly with the mild AD and normal stages (Fig. S3C). The expression level of AβO in the nasal discharge was most correlated with cognitive function in the moderate AD stage. Our results statistically confirmed that the level of specific oligomerized Aβs in the nasal discharge correlated with changes in cognitive function in different AD stages, which was probably due to AD-related dementia progression.

**Table 3:** Correlation analysis between levels of soluble Aβ oligomers and cognitive function.

	Normal—mild	Normal—Moderate	Normal—Mild + Moderate
Self-standard ratio (a.u)	R = 0.5603 (***)	R = 0.3087 (***)	R = 0.5239 (***)
Aβ*56 level (a.u) (ratio of normal)	R = 0.6815 (***)	R = 0.6909 (***)	R = 0.5246 (***)
AβO level (a.u) (ratio of normal)	R = 0.6061 (***)	R = 0.4010 (***)	R = 0.5173 (***)

Correlation analysis between the total levels of soluble Aβ species (Self-standard ratio, Aβ*56 and AβO) in nasal discharges and MMSE scores was conducted. Linear regression analyses of the total oligomeric soluble Aβ showed significant correlation with the MMSE score. We calculated the correlation between soluble Aβ oligomer levels and cognitive function with the line shows the regression line with 95% confidence interval. Statistical significances are denoted (***P < 0.001).

### Longitudinal measurements of cognitive function with high Aβ*56 expression levels in their nasal discharges

**Figure 3:**
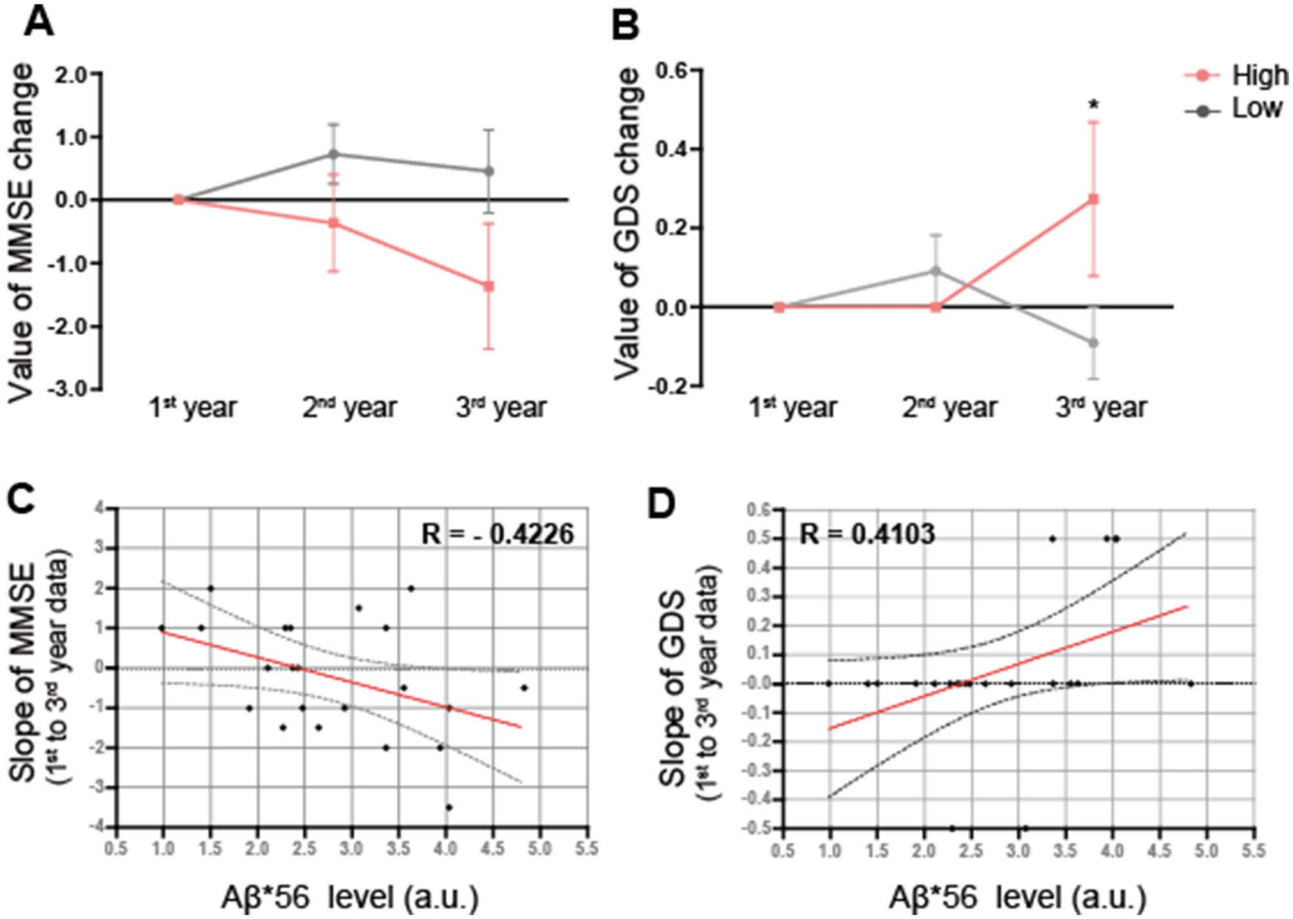
The association between soluble Aβ oligomer levels and cognitive performance over time (for 3 years). (**A**) MMSE change pattern over 3 years (baseline is 1st year). (**B**) GDS change pattern over 3 years (baseline is 1st year). Marginally significance on interaction by two-way RMANOVA (*P* = 0.050). High group show higher change of GDS compare to Low group on 3rd year. Statistical significance is denoted (ns > 0.05 and *P < 0.05) followed by Bonferroni post hoc test. (**C**–**D**) Correlation analysis between soluble Aβ oligomer levels and slope of cognitive performance. Increased levels of soluble Aβ oligomers in nasal discharge are associated with declining cognitive status over time. This effect is constant over time; levels of soluble Aβ oligomers in nasal discharge are significantly associated with declining cognitive status after 3 years.

We performed the longitudinal cohort study of mild AD patients with distinct Aβ*56 expression levels in their nasal discharges over three years. We divided the mild AD group (n = 22) within the total AD subjects into two groups based on their Aβ*56 levels in nasal discharges. We determined the baseline for dividing the groups using the average of Aβ*56 levels in the total AD subjects (n = 38). Since the average Aβ*56 level was 2.65, we set 2.65 as the baseline for dividing the mild AD group (n = 22). The subjects with Aβ*56 levels below 2.65 were grouped as into a Low group (n = 11), whereas participants with Aβ*56 levels above 2.65 were grouped into a High group (n = 11) (Table 4). Then, the changes in the MMES and GDS scores in both groups were monitored annually for three years (46 ± 7 months) (Table 4). We found that the High group experienced a declining trend in MMSE scores (Fig. 3A) and a significant increase in the GDS scores (Fig. 3B) within three years. We also found that the expression level of Aβ*56 in nasal discharge correlated with changes in cognitive function in the AD subjects. When stratified by MMSE score changes in the AD subjects over three years (1st to 3rd-year data), the levels of Aβ*56 were moderately correlated with MMSE scores (R = -0.4226; red line) (Fig. 3C). Similarly, the expression levels of Aβ*56 were also moderately correlated (R = 0.4103; red line) with changes in the GDS scores in AD subjects over three years (1st to 3rd year data) (Fig. 3D). This finding suggests that high Aβ*56 expression levels in their nasal discharges are associated with cognitive decline in AD patients.

**Table 4:** Summary of divided groups by Aß*56 level.

Group	Low (Aß*56 level < 2.65)			High (Aß*56 level ≥ 2.65)		
Age	76.82 ± 4.88			74.45 ± 5.77		
Sex	M		F	M		F
	4		7	3		8
Aß*56 level	2.01 ± 0.48			3.58 ± 0.58		
Test year	1st year	2nd year	3rd year	1st year	2nd year	3rd year
MMSE	21.09 ± 3.34	21.82 ± 3.19	21.55 ± 2.97	18.82 ± 3.21	18.45 ± 4.36	17.45 ± 4.98
GDS	3.27 ± 0.45	3.36 ± 0.48	3.18 ± 0.39	3.55 ± 0.66	3.55 ± 0.66	3.82 ± 0.72

The data are represented as means ± SDs. Each group was divided to include the same number of subjects based on the Aß*56 level. Low group: Aß*56 levels below 2.65, High group: Aß*56 levels above 2.65. MMSE and GDS refer to the Mini-Mental State Examination and the Global Deterioration Scale, respectively.

## Discussion

Declining sensory function is common in neurodegenerative disorders, including AD^27,28^. In particular, olfactory dysfunction is an indisputable characteristic of patients with AD^29,30^. Meta-analysis studies on olfactory function and cognitive dysfunction reported significant problems in an odor processing pathway in presumed and confirmed cases of AD^31^. Therefore, it is highly intriguing to test whether a particular state of olfaction represents AD pathology. The symptoms of olfactory dysfunction have been attributed to neurodegeneration occurring in the olfactory central pathway in the central nervous system^4,32^. However, the findings of the present study that soluble Aβ oligomers were easily detectable in the nasal discharge of the probable AD group may suggest an alternative explanation. Moreover, the finding that the expression profile of soluble Aβ oligomers closely correlated with a decline in the cognitive performance of patients with probable AD pathology may provide an opportunity for the early screening of AD by nasal discharge samples.

**Figure 4:**
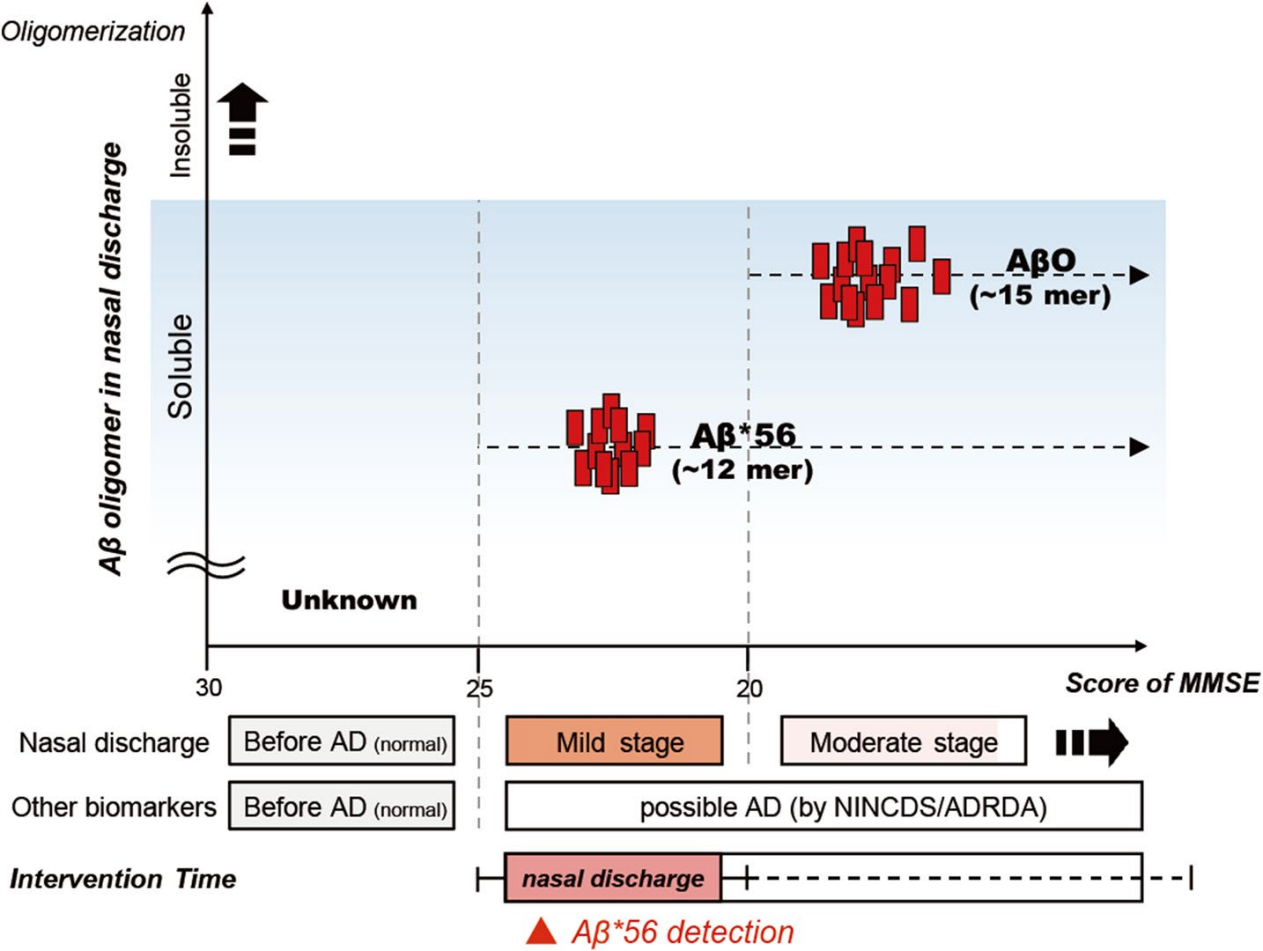
Schematic diagram which laid advantage out of AD diagnosis using nasal discharge.

The peripheral olfactory system contributes to AD-related olfactory dysfunction via its processing of the amyloid precursor protein^9^, although the precise mechanisms remain unclear. Moreover, another study on the peripheral olfactory system in an AD mouse model also showed that the expression of soluble Aβ oligomers, Aβ*56 and AβO in the olfactory system was toxic to olfactory sensory neurons and consequently led to olfactory impairments^16^. Direct links to studies using an AD model mouse of human AD dementia should be made cautiously, however, the results using an AD model mouse were highly comparable to the results using human nasal discharge presented here. The unregulated oligomerization of Aβ in the nasal secretion of patients with AD dementia was also recently reported^33^, although the direct identification of Aβ in nasal discharge, as well as the relative expression of Aβ oligomer isoforms in the nasal discharge, were not shown. Here, we first identified Aβ proteins in human nasal discharge in patients with serious cognitive decline (probably with AD dementia) and the total amount of Aβ proteins increased in the nasal discharge of patients with possible AD dementia. From our qualitative analysis of Aβ in nasal discharge, we revealed two specific types of Aβ oligomers, which were well validated by previous studies in either mice or humans. Through the results, we confirmed that a certain amount of amyloid β can be detected in both the normal and patient groups. However, a specific amyloid beta (Aβ*56, AβO) that can clearly distinguish the normal group from the patient group was tested. In previous study, it has been demonstrated that an oligomerized form of Aβ, Aβ*56, correlated with cognitive deficits and that AβO, a more oligomerized form, induced direct cytotoxicity and significantly mediated cell death during AD progression in a mouse model^34,35^. Taken together, we propose that Aβ*56 is dramatically upregulated in the peripheral olfactory system during the early stages of dementia, followed by an increase in AβO expression during the later stages of AD dementia. Furthermore, we suggest that these observations imply that increases in soluble Aβ aggregates in the peripheral olfactory system may be closely related to AD progression and then, that Aβ aggregation in the peripheral olfactory system may precede diminished cognitive function in the CNS. In fact, our 3-year longitudinal cohort study showed that high levels of Aβ*56 in the nasal discharge in the mild AD group could be a premonitory symptom of the further catastrophic progression of dementia. It is still difficult to claim a direct link between Aβ oligomers in nasal discharge and AD pathogenesis in the brain since such a claim requires an explanation of how the soluble Aβ oligomers in the olfactory system are associated with AD-related cognitive impairment. Despite many reservations, we propose a novel and convenient approach for monitoring cognitive decline with possible AD progression (Fig. 4).

To date, a number of clinical trials have been conducted to overcome progressive neural dysfunction in patients with AD dementia. However, only minor delays in disease progression have been achieved^36–38^. Therefore, treatment effectiveness may be maximized by timely intervention. To this end, a great deal of effort is currently being exerted to optimize the monitoring or either AD initiation or progression. Here, we showed that the levels of soluble Aβ oligomers in nasal discharge were significantly higher in patients with probable AD. Furthermore, routine nasal discharge screenings can be a better option in AD screening due to additional advantages, such as relatively low cost, non-invasive sampling and so on. A few issues still need to be clarified, such as retrospective cross-sectional verification studies of Aβ in the nasal discharge and identification of other biomarker candidates in the nasal discharge. In addition, Relationships with other neurodegenerative diseases by associations in underlying pathological, physiological, and possibly genetic linkages also need to be considered in the selection of other biomarker candidates^39^. In particular, when considering limitations in the accuracy of AD diagnosis known to date^40^, additional experiments considering other neurodegenerative disease patient groups, such as PDs, may be necessary in future plan. However, our results from patients with probable AD reveal the feasibility of using nasal discharge to screen for AD biomarkers, as well as biomarkers to monitor AD progression. Taken together, the results of this study introduce a novel and simple approach to assessing AD progression by monitoring the expression profile of soluble Aβ oligomers in nasal discharge.

## Materials and methods

### Group selection criteria

Patient criteria in this study followed the NINCDS/ADRDA of the American Psychiatric Association criteria for the diagnosis of probable AD^1,21–23^.

Briefly, samples from the patients were divided into three groups based on the following criteria. Patients with probable AD was selected according to the following criteria: (1) predominant and progressive episodic memory impairment characterized by low free recall, not normalized to cueing and not associated with other cognitive deficits; and (2) scores of the MMSE, the CDR, and the GDS tests that are commonly applied to measure the severity of dementia from various causes^1,2,21^. All tests of cognitive ability were analyzed as previously described^41^. Briefly, the Korean version of the MMSE is comprised of tests of orientation (10 points), short-term memory registration and recall (6 points), attention (5 points), naming (2 points), following verbal commands (4 points), judgment (2 points), and copying a double pentagon (1 point). The CDR scale is a structured interview of the subject and informant in which the subjects are rated by scores of 0 (asymptomatic), 0.5 (equivocal or mild impairment), 1, 2 or 3 (mild, moderate, or severe dementia, respectively). The normal group had MMSE scores greater than 27 and normal scores in the memory section of the MMSE. The probable AD patient group had scores of 24 or less on the MMSE and CDR scores (1) greater than 1 or (2) 0.5 with box score sums greater than 2.5. The GDS scale is one of the most popular scales for the evaluation of depression symptoms in older adults. In the long form, a score is considered normal if it is between 0 and 0.9. An indicator of mild depression is between 1.0 and 1.9 and a positive score for severe depression is between 2.0 and 3.0. Patients with other neurodegenerative diseases, such as Parkinson’s disease; cerebral vascular disease (which may affect cognitive function); metabolic derangement, including thyroid disease, a history of alcohol or medication poisoning; or a history of trauma or neuropsychiatric disease were excluded from the current study.

### Measurement of Aβ levels in nasal discharge using an IME sensor (an impedimetric biosensor with an interdigitated microelectrode structure)

An IME biosensor with 30 pairs of interdigitated microelectrodes with 5 µm-wide gaps was utilized for this analysis^42,43^. The gap surface was functionalized with 6E10 antibodies to capture the Aβ protein from nasal discharge samples. The fabrication, antibody immobilization, and detection procedures are described in our previous study^27^. When a specific interaction occurred between 6E10 on the sensor surface and an Aβ peptide, the impedance value between the interdigitated microelectrodes was altered because the Aβ protein occupied the space instead of the fluid. To perform the CLASS method, the nasal discharge samples were aliquoted into two samples. For measuring the EPPS-treated sample and PBS-added sample, 40 µL of each sample was used. One sample was incubated for 30 min with 500 mM EPPS [4-(2-hydroxyethyl)-1-piperazinepropanesulfonic acid] dissolved in phosphate-buffered saline (PBS) (nasal discharge sample: EPPS, 4:1) and the other sample was treated the same with the exclusion of EPPS. The prepared nasal discharge sample was injected onto individual IME devices, incubated for 20 min, and washed with PBS buffer. Then, the impedance of 6E10-immobilized IME before (Z_before_) and after (Z_after_) the reaction of nasal discharge containing Aβ proteins was measured. The impedance of the impedimetric biosensors was measured using commercial equipment (PGSTAT302N, Metrohm Autolab, Utrecht, The Netherlands; & IME Sensor, Cantis Corporation, Ansan, Korea). The impedance change was used to quantify the Aβ levels in nasal discharge which was defined by the equation below.

$$Impedance \,change\, \left( \% \right) = \left| {\frac{{Z_{after} - Z_{before} }}{{Z_{before} }}} \right| \times 100$$

The self-standard ratio defined the value of the impedance change in the EPPS-treated sample (monomerized) divided by the impedance change in the PBS buffer-added sample.

### Statistical analysis

The results were presented as mean ± SEM. Value of samples were identified as outliers through Grubbs’ test, also called the ESD method. The Kolmogorov–Smirnov test, paired t-test, one-way analysis, and two-way RMANOVA of variance were used to assess the normality of the data. The nonparametric Spearman's rank correlation test was used to assess correlations between the data. The graphs revealed regression lines with a 95% confidence interval. *P* values of < 0.05 were considered significant. Cognitive function changes were measured by MMSE and GDS scores over three years in AD subjects, defined by the equation below.

$$Slope = \frac{{h_{3} - h_{1} }}{{L_{3} - L_{1} }}$$

(h = MMSE score or GDS score, L = year).

### Study approval

The Institutional Review Boards (IRB) of Gachon University Gil Medical Center [GAIRB2013-264] approved the study protocol. All subjects provided written informed consent before participating via self-referral or referral from their family.

### Nasal discharge collection and processing

Nasal discharge samples from 60 donors were analyzed. Twenty additional samples were collected but excluded from the analysis due to insufficient protein concentration (n = 8) or insufficient sample for three independent WB and IME sensor analyses (n = 12). Age-matched normal subjects (n = 21) and patients with probable AD (n = 39) were also assessed. The details of each group are presented in Table 1.

The whole nasal discharges were pooled (> 1.5 ml) in a microtube and immediately sonicated for 10–15 s, followed by centrifugation (10,000xg for 10 min at 4 °C) to remove cells and cellular debris. A Protease Inhibitor Cocktail was added to the supernatants (Roche, Mannheim, Germany), which were immediately stored at −80 °C until analysis. Nasal discharge aliquots were thawed on the day of the experiment.

### Liquid chromatography-mass spectrometry/mass spectrometry (LC–MS/MS) analysis

The immunoprecipitation and immunoblots was modified and performed as described previously^44^. For immunoprecipitation, aliquots of human nasal discharge samples (300 µl) were pre-cleared with 30 µl of Protein-G Fast Flow Sepharose (GE Healthcare Life Sciences, Uppsala, Sweden) for 1 h at 4 °C, then centrifuged at 9300 g for 5 min. Subsequently, 250 µl of immunoglobulin-depleted nasal discharge was incubated with 1 µg of 6E10 antibodies (6E10, Covance, Princeton, NJ, USA) and 50 µl of Protein-G coated magnetic beads (Life Technologies, CA, USA) overnight at 4 °C. The beads were washed sequentially with immunoprecipitation buffer A [50 mM Tris–HCl, 300 mM NaCl, 0.1% Triton X-100 (v/v), 1 mM EDTA, pH 7.4] and immunoprecipitation buffer B [50 mM Tris–HCl, 150 mM NaCl, 0.1% Triton X-100 (v/v), 1 mM EDTA, pH 7.4] for 20 min under gentle agitation at 4 °C. Next, the captured proteins were eluted and digested with trypsin. Initially, sample reduction was conducted using 20 mM dithiothreitol for 1 h and alkylated with 55 mM iodoacetamide for 45 min. Trypsin digestion was carried out overnight using mass spectrometry-grade TPCK-treated small trypsin (ABSciex**,** Framingham, MA, USA). The stabilized, digested peptides were extracted and lyophilized. Before LC–MS / MS analysis, the peptide samples were resuspended in 10 μl of 1% formic acid.

Prior to mass spectrometry, the peptides were separated using EasynLCII (Bruker Daltonics, Bremen, Germany) nano high-performance liquid chromatography (HPLC) for intervals of at least 60 min after using water/acetonitrile gradient with increases in acetonitrile concentrations from 0 to 100% for 90 min. The peptide mixture was desorbed on a Zorbax 300SB-C18 analytical column (150 mm × 75 μm 3.5 μm pore size, Agilent, Santa Clara, CA, USA) after desalination on a Zorbax 300SB-C18 inline trap column (5 × 0.3 mm, 5 μm pore size, Agilent). Solvent A was 0.1% formic acid in LC/MS Grade water, solvent B was LC/MS Grade acetonitrile containing 0.1% formic acid, and the flow rate was 300 nl/min.

The obtained LC–MS/MS data were used to search for matches in the SwissProt database (release: 2015.07, 548,872 sequence item) using the ProteinPilot 4.0 (AB SCIEX, Framingham, MA) search engine and to identify proteins using the biological variation tables included in the ProteinPilot 4.0 software (Fig. S1A).

### Immunoprecipitation and immunoblots

The immunoprecipitation and immunoblots was modified and performed as described previously^45^. For immunoprecipitation with 6E10 and immunoblotting with the A11 antibody, aliquots of the samples (100 µl) were pre-cleared with 30 µl of a 1:1 slurry with Protein-G Fast Flow Sepharose (GE Healthcare Life Sciences, Uppsala, Sweden) for 1 h at 4 °C, then centrifuged at 9300 g for 5 min. Subsequently, 250 µl of immunoglobulin-depleted nasal discharge was incubated with 0.1 µg of 6E10 antibodies and 50 µl of Protein-G coated magnetic beads (Life Technologies, CA, USA) overnight at 4 °C. The beads were washed sequentially with immunoprecipitation buffer A [50 mM Tris–HCl, 300 mM NaCl, 0.1% Triton X-100 (v/v), 1 mM EDTA, pH 7.4] and immunoprecipitation buffer B [50 mM Tris–HCl, 150 mM NaCl, 0.1% Triton X-100 (v/v), 1 mM EDTA, pH 7.4] for 20 min under gentle agitation at 4 °C. The captured proteins were eluted with SDS-sample buffer. The proteins were separated by sodium dodecyl sulfate–polyacrylamide gel electrophoresis and transferred to a 0.45-μm polyvinylidene difluoride membrane (Millipore, Temecula, CA, USA). The membranes were blocked with 5% non-fat dry milk in Tris-buffered saline with 0.1% Tween 20 and then incubated with primary A11 (Invitrogen, Carlsbad, CA, USA) antibodies for oligomerized Aβ proteins and 6E10 (Covance, Princeton, NJ, USA) for total Aβ proteins.

For immunoblotting with the A11, D54D2 and 6E10 antibody, the nasal discharge was thawed and the proteins were quantified by BCA assay. Then 5 μg of protein were separated by sodium dodecyl sulfate–polyacrylamide gel electrophoresis and transferred to a 0.45-μm polyvinylidene difluoride membrane (Millipore, Temecula, CA, USA). The membranes were blocked with 5% non-fat dry milk in Tris-buffered saline with 0.1% Tween 20 and then incubated with primary antibodies, A11 (Invitrogen, Carlsbad, CA, USA) and 6E10 (Covance, Princeton, NJ, USA). The immunoblots were visualized using a commercial development kit (Pierce, Dallas, TX, USA). Quantification of the immunoblots was performed using the ImageJ program (NIH, USA). The normalization of the data was performed by dividing the quantified value of protein by the total protein amount.

### Ethics declarations

All methods were carried out in accordance with relevant guidelines and regulations.

## Supplementary information

Supplementary Figures S1 - S4

## Abbreviations

AD: Alzheimer’s disease
CDR: Clinical dementia rating
Aβ: Amyloid-β
Aβ42: Amyloid-β 1–42 peptide
CSF: Cerebrospinal fluid
ELISA: Enzyme-linked immunosorbent assay
GDS: Global deterioration scale
HRP: Horseradish peroxidase
MDS: Multimer detection system
MMSE: Mini mental state examination
MRI: Magnetic resonance imaging
PBS: Phosphate-buffered saline
RLU: Relative light/luminescence units
TBST: Tris-buffered saline with Tween 20
