# Supplementary material for: Longitudinal profiling of oligomeric Aβ in human nasal discharge reflecting cognitive decline in probable Alzheimer’s disease
Source: Sci Rep. 2020 Jul 8;10:11234. doi: 10.1038/s41598-020-68148-2 (PMC7343787; doi:10.1038/s41598-020-68148-2)
Supplement: Supplementary file 1 — Supplementary Figures S1 - S4 [file 41598_2020_68148_MOESM1_ESM.pdf]

Supplement materials:

Longitudinal profiling of oligomeric Aβ in human nasal discharge reflecting cognitive decline in probable Alzheimer's disease

Seung-Jun Yoo[1,2,#], Gowoon Son[1,#], Jisub Bae[1], So Yeun Kim[1,2] Yong Kyoung Yoo[3], Dongsung Park[3], Seung Yeop Baek[4], Keun-A Chang[5], Yoo-Hun Suh[5], Yeong-Bae Lee[6], Kyo Seon Hwang[3], YoungSoo Kim[4], Cheil Moon[1,2,*.]

[1]Department of Brain & Cognitive Sciences, Graduate School and [2]Convergence Research Advanced Centre for Olfaction, Daegu Gyeungbuk Institute of Science and Technology, Daegu, Republic of Korea, [3]Department of Clinical Pharmacology and Therapeutics, College of Medicine, Kyung Hee University, Seoul, Republic of Korea, [4]Integrated Science and Engineering Division, Department of Pharmacy, and Yonsei Institute of Pharmaceutical Sciences, Yonsei University, Incheon, Republic of Korea, [5]Department of Pharmacology, School of Medicine, Gachon Medical School, Incheon, Republic of Korea, [6]Department of Neurology, Gil Medical Center, Gachon University, Incheon, Republic of Korea.

[#]These two authors contributed equally to this work

[*]**Correspondence:** Cheil Moon, Ph.D., Department of Brain & Cognitive Sciences, Graduate School, Daegu Gyeungbuk Institute of Science and Technology, 333, Techno Jung-Ang Daero, Hyeonpung-Myeon, Dalseong-Gun, Daegu, 711-873, Korea. E-mail: cmoon@dgist.ac.kr; Tel: +82-53-785-1040; Fax: +82-53-785-6109

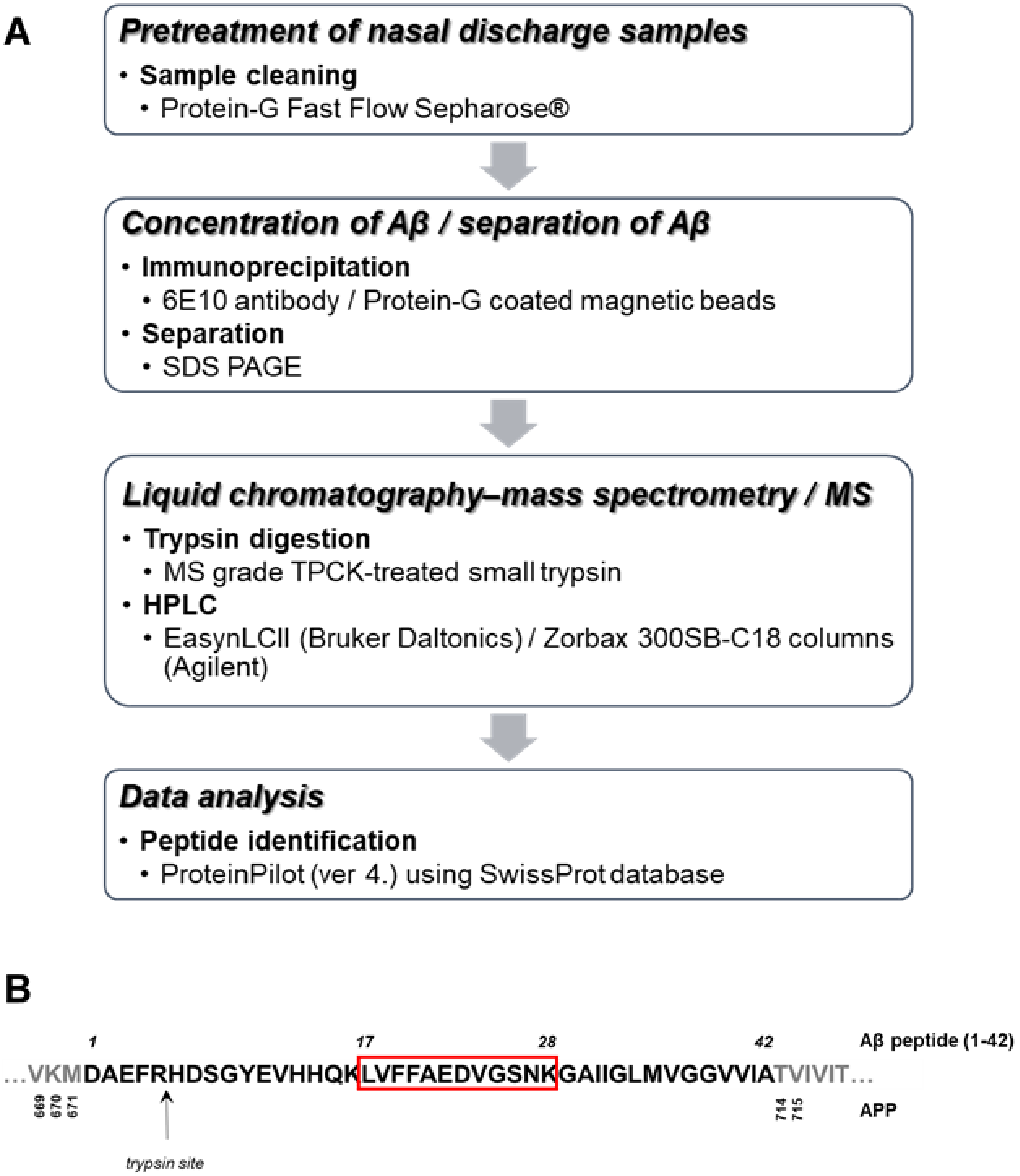
A
Pretreatment of nasal discharge samples
• Sample cleaning
• Protein-G Fast Flow Sepharose®
Concentration of Aβ / separation of Aβ
• Immunoprecipitation
• 6E10 antibody / Protein-G coated magnetic beads
• Separation
• SDS PAGE
Liquid chromatography–mass spectrometry / MS
• Trypsin digestion
• MS grade TPCK-treated small trypsin
• HPLC
• EasynLCII (Bruker Daltonics) / Zorbax 300SB-C18 columns (Agilent)
Data analysis
• Peptide identification
• ProteinPilot (ver 4.) using SwissProt database
B
1
17
28
42
Aβ peptide (1-42)
…VKMDAEFRHDSGYEVHHQKLVFFAEDVGSNKGAIIGLMVGGVVIATVIVIT…
669
670
671
714
715
APP
trypsin site

Supplement Fig. 1. Identification and quantification; soluble Aβ oligomers were detected in the nasal discharge of patients with AD. (A) Proteomic identification of soluble Aβ oligomers by immunoprecipitation (IP). Schematic of the workflow of profiling of soluble Aβ oligomers by IP combined with LC-MS/MS. (B) Sequence of Aβ1−42, with the cleavage sites for trypsin (arrows at the bottom of the sequence). Identified sequence is belonged to Aβ sequence.

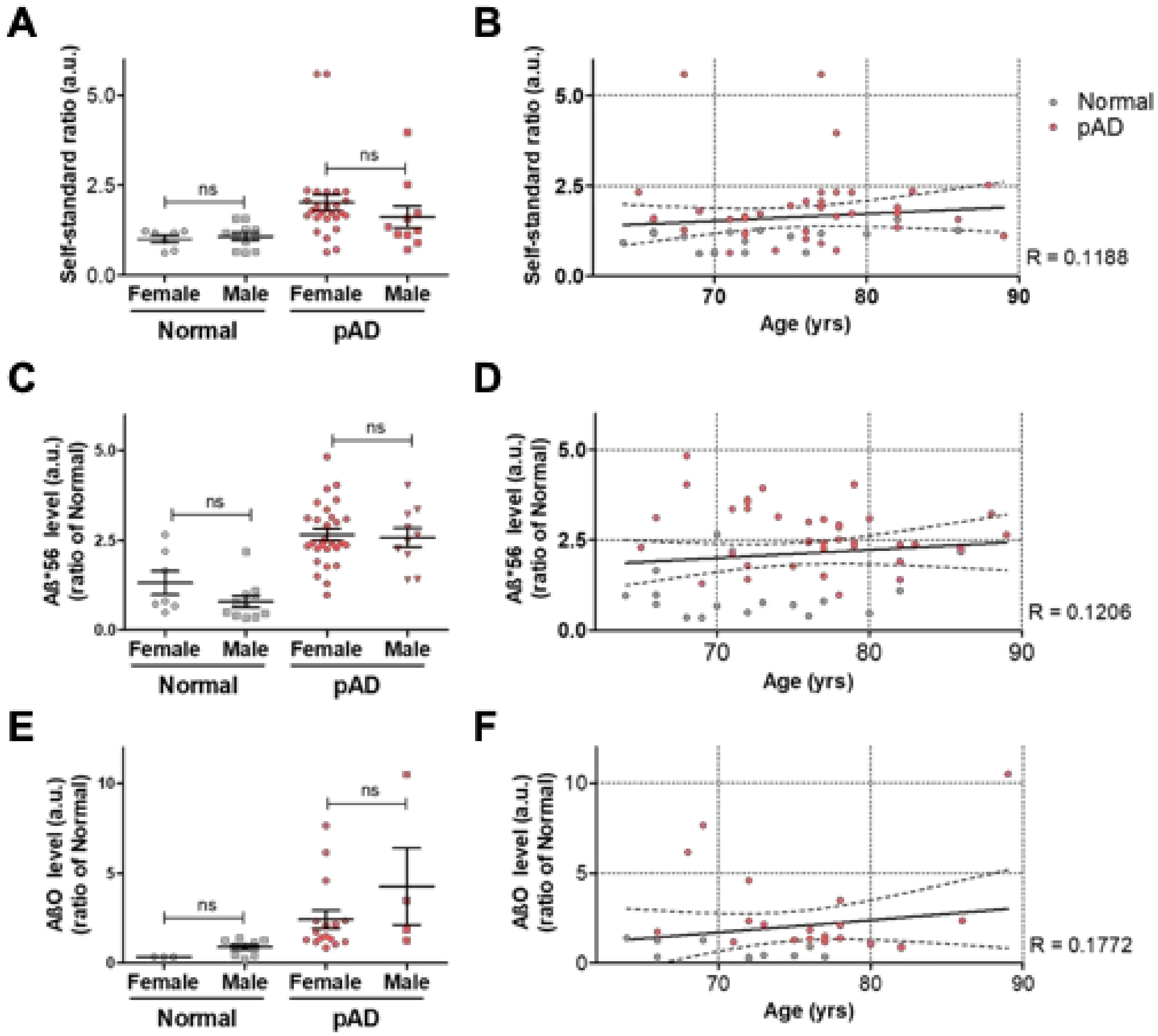
A
Self-standard ratio (a.u.)
5.0
2.5
0.0
ns
ns
Female
Male
Female
Male
Normal
pAD
B
Self-standard ratio (a.u.)
Normal
pAD
R = 0.1188
70
80
90
Age (yrs)
C
Aβ*56 level (a.u.)
(ratio of Normal)
ns
ns
Female
Male
Female
Male
Normal
pAD
D
Aβ*56 level (a.u.)
(ratio of Normal)
R = 0.1206
Age (yrs)
E
AβO level (a.u.)
(ratio of Normal)
10
5
0
ns
ns
Female
Male
Female
Male
Normal
pAD
F
AβO level (a.u.)
(ratio of Normal)
R = 0.1772
Age (yrs)

Supplement Fig. 2. Non-significant correlation between soluble Aβ oligomer and age/gender. The total levels of soluble Aβ species using CLASS method (self-standard ratio (a.u.)) were analyzed by gender (A) and age (B). (B) Linear regression analyses showed no significant effects of storage time on biomarker concentrations. ($R = 0.1188$). Quantification of soluble Aβ *56 protein was analyzed by gender (C) and age (D). (D) Linear regression analyses showed no significant effects of age on biomarker concentrations. ($R = 0.1206$). Quantification of soluble AβO protein was analyzed by gender (E) and age (F). (F) Linear regression analyses showed no significant effects of age on biomarker concentrations. ($R = 0.1772$). (A), (C), (E) Data are represented as means ± SEMs from three independent experiments. For statistical analysis, paired t-test was performed. Statistical significances are denoted. (ns; $P > 0.05$) (B), (D), (F) Linear regression analyses shows significant correlation between levels of the total levels of soluble Aβ species using CLASS method (self-standard ratio (a.u.)) (B), Aβ *56 protein (D) and AβO protein (F) with the age(yrs). The line shows the regression line with 95% confidence interval (dashed line).

A

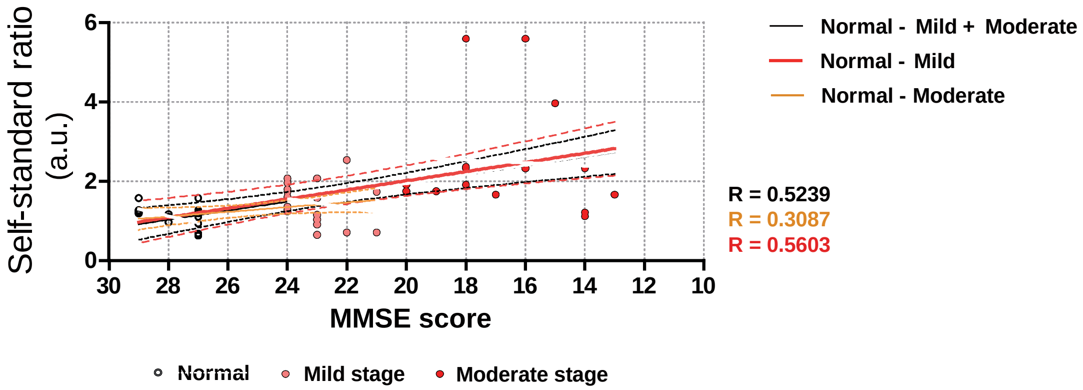

Self-standard ratio (a.u.)
6
4
2
0
30 28 26 24 22 20 18 16 14 12 10
MMSE score
Normal - Mild + Moderate
Normal - Mild
Normal - Moderate
R = 0.5239
R = 0.3087
R = 0.5603
Normal
Mild stage
Moderate stage

B

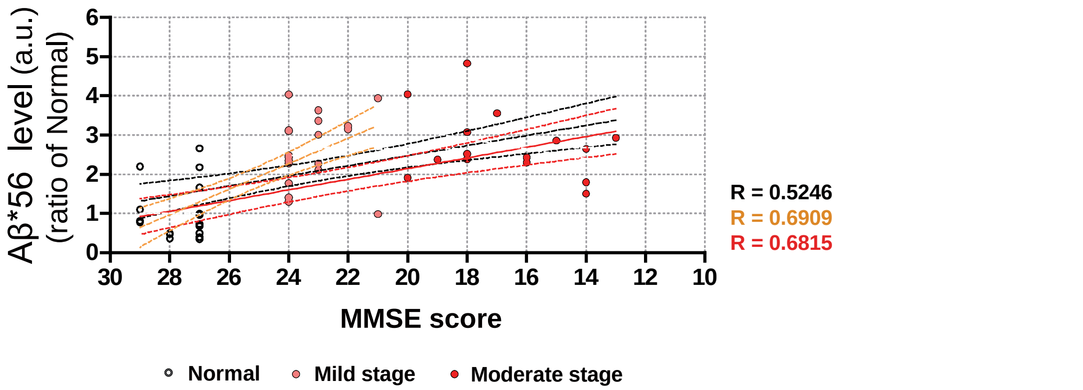

Aβ*56 level (a.u.) (ratio of Normal)
6 5 4 3 2 1 0
30 28 26 24 22 20 18 16 14 12 10
MMSE score
R = 0.5246
R = 0.6909
R = 0.6815
Normal
Mild stage
Moderate stage

C

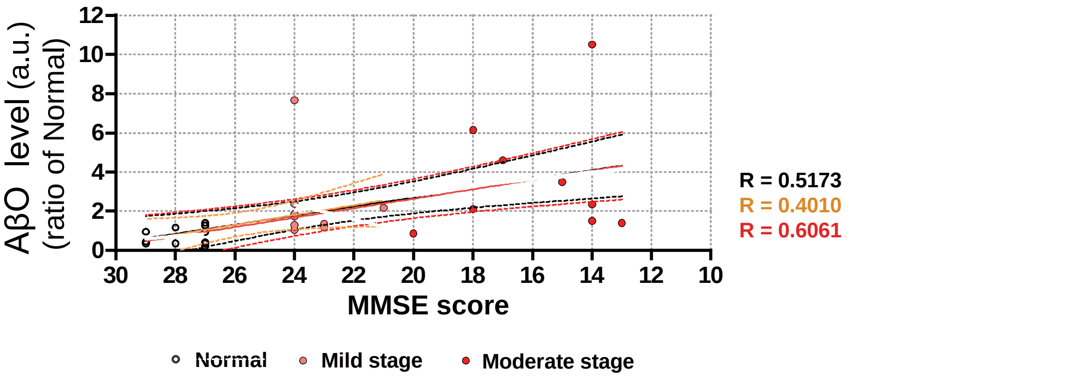

AβO level (a.u.) (ratio of Normal)
12 10 8 6 4 2 0
30 28 26 24 22 20 18 16 14 12 10
MMSE score
R = 0.5173
R = 0.4010
R = 0.6061
Normal
Mild stage
Moderate stage

Supplement Fig. 3. Correlation analysis between levels of soluble Aβ oligomers and cognitive function. (A) Correlation analysis between the total levels of soluble Aβ species in nasal discharges and MMSE scores was conducted. Linear regression analyses of the total oligomeric soluble Aβ showed significant correlation with the MMSE score. (B) Correlation analysis between Ratio of Aβ *56 protein and MMSE scores was conducted. Linear regression analyses of Aβ *56 protein showed significant correlation with the MMSE score. (C) Correlation analysis between Ratio of AβO protein and MMSE scores was conducted. Linear regression analyses of AβO protein showed significant correlation with the MMSE score. Scatter diagrams displaying correlation between soluble Aβ oligomer levels and cognitive function. The line shows the regression line with 95% confidence interval (dashed line).

A

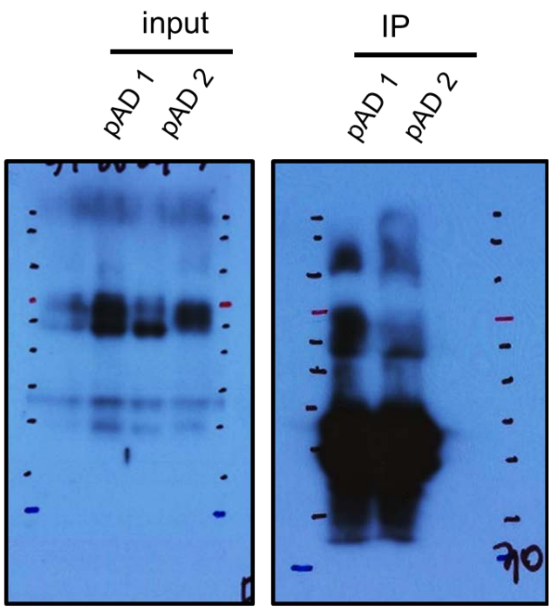
input
IP
pAD 1
pAD 2
pAD 1
pAD 2
70

B

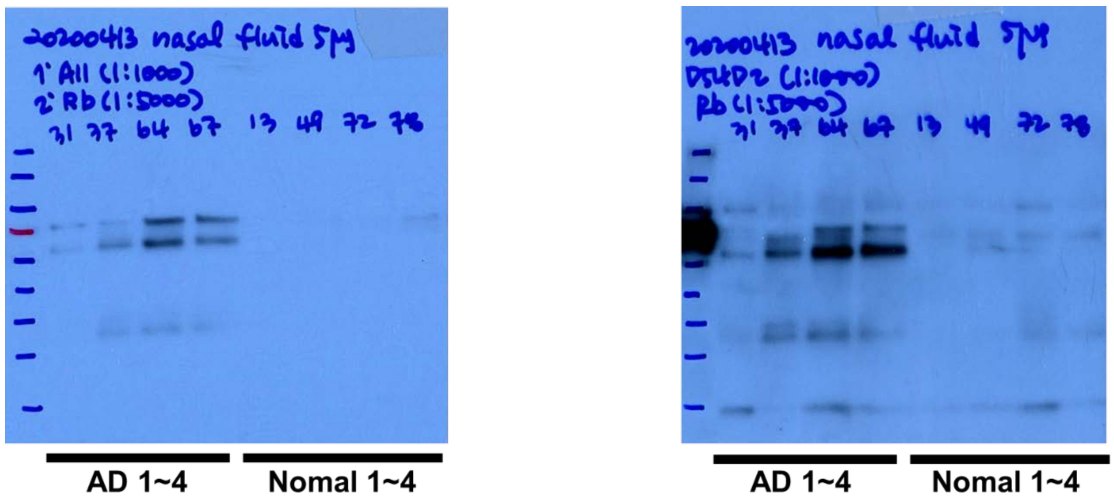
20200413 nasal fluid 5μg
1' All (1:1000)
2' Rb (1:5000)
31 77 64 67 13 49 72 78
AD 1~4
Nomal 1~4
20200413 nasal fluid 5μg
D54D2 (1:1000)
Rb (1:5000)
31 77 64 67 13 49 72 78
AD 1~4
Nomal 1~4

Supplement Fig. 4. Full-size gels and blots for figure 1 (A) and (B).
